# A Carnivorous Plant Fed by Its Ant Symbiont: A Unique Multi-Faceted Nutritional Mutualism

**DOI:** 10.1371/journal.pone.0036179

**Published:** 2012-05-09

**Authors:** Vincent Bazile, Jonathan A. Moran, Gilles Le Moguédec, David J. Marshall, Laurence Gaume

**Affiliations:** 1 Université Montpellier II, UMR AMAP: Botanique et bioinformatique de l'architecture des plantes, Montpellier, France; 2 INRA, UMR AMAP: Botanique et bioinformatique de l'architecture des plantes, Montpellier, France; 3 CNRS, UMR AMAP: Botanique et bioinformatique de l'architecture des plantes, Montpellier, France; 4 School of Environment and Sustainability, Royal Roads University, Victoria, British Columbia, Canada; 5 Biology Department, University of Brunei Darussalam, Gadong, Brunei Darussalam; Stanford University, United States of America

## Abstract

Scarcity of essential nutrients has led plants to evolve alternative nutritional strategies, such as myrmecotrophy (ant-waste-derived nutrition) and carnivory (invertebrate predation). The carnivorous plant *Nepenthes bicalcarata* grows in the Bornean peatswamp forests and is believed to have a mutualistic relationship with its symbiotic ant *Camponotus schmitzi*. However, the benefits provided by the ant have not been quantified. We tested the hypothesis of a nutritional mutualism, using foliar isotopic and reflectance analyses and by comparing fitness-related traits between ant-inhabited and uninhabited plants. Plants inhabited by *C. schmitzi* produced more leaves of greater area and nitrogen content than unoccupied plants. The ants were estimated to provide a 200% increase in foliar nitrogen to adult plants. Inhabited plants also produced more and larger pitchers containing higher prey biomass. *C. schmitzi*-occupied pitchers differed qualitatively in containing *C. schmitzi* wastes and captured large ants and flying insects. Pitcher abortion rates were lower in inhabited plants partly because of herbivore deterrence as herbivory-aborted buds decreased with ant occupation rate. Lower abortion was also attributed to ant nutritional service. The ants had higher δ^15^N values than any tested prey, and foliar δ^15^N increased with ant occupation rate, confirming their predatory behaviour and demonstrating their direct contribution to the plant-recycled N. We estimated that *N. bicalcarata* derives on average 42% of its foliar N from *C. schmitzi* wastes, (76% in highly-occupied plants). According to the Structure Independent Pigment Index, plants without *C. schmitzi* were nutrient stressed compared to both occupied plants, and pitcher-lacking plants. This attests to the physiological cost of pitcher production and poor nutrient assimilation in the absence of the symbiont. Hence *C. schmitzi* contributes crucially to the nutrition of *N. bicalcarata*, via protection of assimilatory organs, enhancement of prey capture, and myrmecotrophy. This combination of carnivory and myrmecotrophy represents an outstanding strategy of nutrient sequestration.

## Introduction

Ant-plant mutualisms play key roles in the functioning of tropical ecosystems, and are often important components of trophic webs [1], [2] but the net benefits to each partner are rarely quantified [3]. The ants usually receive food rewards from the plants in the form of sugar exudates from extra-floral nectaries, or sap-sucking homopterans [4]. If the plant partner is a myrmecophyte, the ants also often benefit from food bodies rich in proteins and/or lipids, as well as from specialised nesting structures, called domatia [5]. Although they are less obvious and less easily quantifiable, three types of benefits to the plant are conferred by ants: seed dispersion, protection against phytophagous insects, pathogenic fungi and/or encroaching vines, and nutrition [6]. The nutritional service is known as myrmecotrophy [7], [8], and occurs when plants assimilate the decomposition products of ants' feces and other organic debris accumulated within the domatia. Food exchange has been demonstrated directly using enriched isotopic tracing (e.g. [9]–[11]), and indirectly by natural abundance isotopic modelling (e.g. [12], [13]). Recently, it has been shown that the nutritional service provided by ants to host plants in some systems is mediated by specific fungi tended inside the domatia [14], [15].

Myrmecotrophic plants have thus evolved mutualistic relationships with ants and are generally encountered in ecosystems where nitrogen (N) is scarce or unavailable, for instance, in temporarily flooded or epiphytic habitats [8], [16]. Carnivorous plants often grow in the same nutrient-poor habitats [16] but have developed a predatory strategy devoted to the assimilation of insect-derived nutrients [17]–[19].

**Figure 1:**
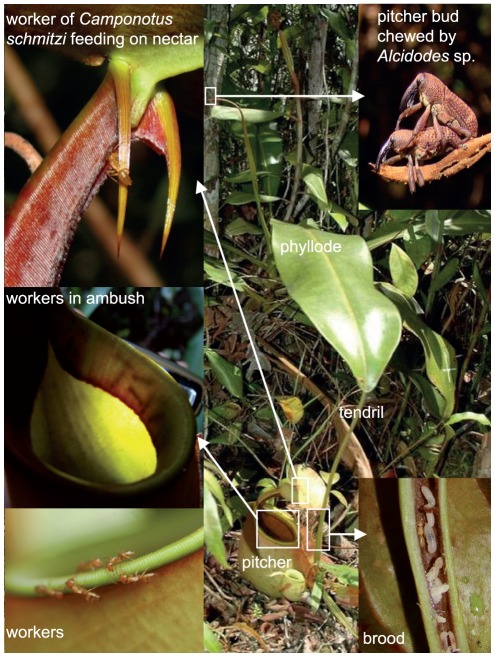
A juvenile *Nepenthes bicalcarata* showing leaf structure (photosynthetic phyllode, tendril and trap [here a lower pitcher]). The upper left inset shows a worker of *Camponotus schmitzi* feeding on nectar from one of the two “fangs” characterizing *N. bicalcarata*. The middle left inset shows workers of *C. schmitzi* in ambush beneath the peristome. The lower insets show workers (left) and brood (right) of *C. schmitzi*, respectively, on the peristome and in the hollow and swollen apical part of the tendril, i.e. the domatium. The upper right inset shows the *Nepenthes*-specific weevil, *Alcidodes* sp. copulating and feeding on a pitcher bud.

*Nepenthes bicalcarata* Hook. f. (Nepenthaceae) produces large photosynthetic blades, or phyllodes (Fig. 1). Each usually ends in a tendril that bears a carnivorous non-photosynthetic organ, the pitcher, which has a very long life span (*ca.* 9 mo), compared to other *Nepenthes* species [20]. The digestive fluid harbours a community of mostly dipteran and bacterial ‘infauna’ that facilitates prey breakdown [21], [22]. Like most *Nepenthes* species, *N. bicalcarata* exhibits heteroblastic development characterized by ontogenetic pitcher dimorphism with terrestrial (lower) pitchers associated with the young free-standing life stage of the plant, and aerial (upper) pitchers of different shape associated with the mature climbing stage [23], [24].

*N. bicalcarata* is unique among the 120 species in the genus [20], [25] in being a myrmecophyte [26]. At the base of each pitcher, a swollen tendril forms a domatium inhabited specifically by the ant *Camponotus schmitzi* Stärke (Formicidae, Formicinae) ([27] Fig. 1). These plant-ants have never been found in host-plants other than *N. bicalcarata*. They feed on nectar secreted by the teeth of the pitcher rim including the paired, giant “fanged” nectaries (Fig. 1) [28]. Additionally, they consume part of the prey that they help to catch and remove from the digestive fluid [26], owing to an unusual and specific swimming behaviour [29]. Prey are consumed under the peristome (pitcher rim) where the ants spend most of their time; the undigested remains, as well as the ants' feces, are eventually dropped into the pitcher fluid [26]. Thus, in addition to housing, the ants receive carbohydrates and animal proteins from the host plant.

The services provided by *C. schmitzi* in this interaction have yet to be completely elucidated. To date, four non-exclusive hypotheses have been proposed. The first is that the ant protects the pitcher from putrefaction [29]. By removing large prey items that would otherwise release large amounts of ammonia into the pitcher fluid, the ants prevent the death of the associated pitcher fluid inhabitants (infauna) and the disruption of the “digestive” system. The second hypothesis is that *C. schmitzi* provides a more typical “anti-herbivore” benefit, by selectively attacking a *Nepenthes*-specific weevil (*Alcidodes* sp., Curculionidae), which would otherwise feed on and destroy developing pitcher buds (Fig. 1) [30]. Thirdly, it has been demonstrated recently that *C. schmitzi* plays a role in prey capture by the host plant [26]. *N. bicalcarata* lacks both a slippery waxy layer and a viscoelastic fluid [31], two fundamental features of the *Nepenthes* trap [24], [32], [33]. The ants were shown to lie in ambush under the pitcher rim and to attack insect visitors systematically once they fell into the pitcher. The presence of *C. schmitzi* significantly increases the number of insects retained by the pitchers [26]. However, since the ants also consume part of the prey, there remains uncertainty regarding any nutritional benefit to the host plant. Most recently, it has been shown that *C. schmitzi* cleans the peristome, the wettable, slippery collar of tissue surrounding the pitcher mouth [34]. Surface contaminants reduce the trapping efficiency of the peristome, and it has been demonstrated that *C. schmitzi* actively maintains the structure at optimum efficiency by removing fungal and other material [35].

However, despite a body of evidence for the mechanisms underlying the mutualism, there remains the question of measurable physical and/or physiological benefits to *N. bicalcarata* itself. In this study, we investigated whether the relationship between *N. bicalcarata* and *C. schmitzi* represents a functional mutualism. First, we assessed the effect of the ants' presence on growth and photosynthetic area. Long-term experimental exclusion of *C. schmitzi* was impossible to effect without also adversely influencing visits by potential crawling prey; instead, we assessed the effect of *C. schmitzi* presence on leaf area and foliar N content, by comparing naturally-occurring ant-occupied plants (hereafter called PA plants, i.e. Pitcher- and Ant- bearing plants) and unoccupied plants and by comparing plants differing in their degree of ant occupation. We used foliar reflectance analysis [36]–[38] to compare the degree of nutrient stress between plants with or without *C. schmitzi*. We also assessed the cost of carnivory in this plant species by comparing growth and physiology of unoccupied pitcher-producing (PnoA, i.e. Pitchers and no Ant) and pitcher-lacking (NoP, i.e. No Pitcher) plants. We then tested the hypothesis of nutritional mutualism by assessing whether the ants contribute to the plant's nutrition. To this effect, we tested if *C. schmitzi* had a positive impact on prey quantity, by comparing pitcher production, pitcher morphology and associated prey biomass between occupied and unoccupied plants. We investigated whether anti-herbivore protection may partly explain such a pattern. Finally, using natural abundance stable isotope ratios (δ^15^N), we established the trophic position of *C. schmitzi* with regards to the plant's prey and tested the hypothesis of myrmecotrophy by comparing the foliar isotopic signatures of PA and PnoA plants [39], [40]. We then quantified the ant-waste contribution to the N budget of the plant using stable isotope modelling [41], [42].

## Results

### Positive effect of *C. schmitzi* on *N. bicalcarata*'s growth performance

#### Plants inhabited by *C. schmitzi* have greater total leaf area and N content

**Figure 2:**
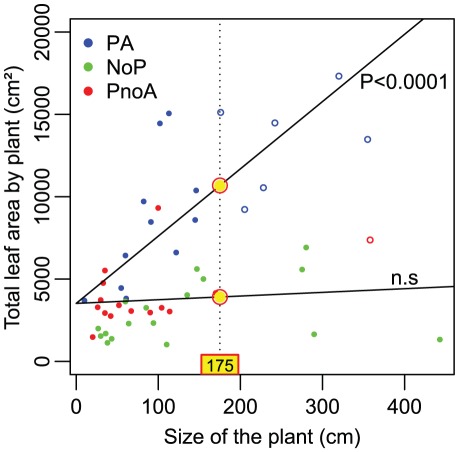
Growth improvement conferred by *Camponotus schmitzi* to its host-plant *Nepenthes bicalcarata*. While the total leaf area increased linearly with plant size for plants occupied by *C. schmitzi* (in blue), this was not the case for unoccupied plants. A common non-significant regression line was estimated for pitcher-bearing (green) and pitcher-lacking (red) unoccupied plants. Filled points are plants which have produced only lower pitchers; empty points are plants which have developed upper pitchers. 175cm is the size threshold at which occupied plants begin to enter the climbing phase. At this threshold, the total leaf area of occupied plants was estimated to be 2.9 times greater than that estimated for unoccupied plants; total leaf nitrogen content was estimated to be 3.3 times greater.

Plants occupied by *C. schmitzi* (PA) had a significantly higher total leaf area (with a presumably greater ability to photosynthesise) than unoccupied plants (PnoA+NoP; pooled together because the model estimated both common slope and common intercept for the regression lines of both groups, ANCOVA, type effect, Table 1, Fig. 2). In contrast to unoccupied plants, the total leaf area of occupied *N. bicalcarata* increased significantly with plant size (significant interaction size*type, Table 1). More precisely, leaf area increased linearly with both plant size and *C. schmitzi* occupation rate (Table 2). For plants 175 cm in height (the size threshold at which an occupied plant enters the climbing phase, Fig. 2), the model estimated a total foliar area of 10964.72±801.55 cm^2^ for a PA plant, compared to only 3767.93±402.21 cm^2^ for an unoccupied plant (a 2.9-fold reduction). Plants occupied by *C. schmitzi* also bore a greater number of leaves than unoccupied plants of the same size (Poisson regression model: type significant, Table 3) and their number of leaves increased with plant size (size effect significant) but at a rate not significantly higher (size*type interaction not significant χ^2^ = 1.82, P = 0.40 and thus removed from the model) than that of unoccupied plants for which the least-squares means were not significantly different between pitcher-bearing and pitcher-lacking plants (PnoA/NoP, χ^2^ = 1.10, P = 0.29).

**Table 1:** Results of ANCOVA testing for the effects of plant size, plant type (PA, PnoA and NoP) and their interaction on total leaf area.

Explanatory variables		*ndf*	*ddf*	*F*	*P*	Estimate	SE
Size		1	43	19.40	<0.0001		
Type		2	43	2.61	0.0850		
Size*type		2	43	5.71	0.0063		
**Parameter estimates**							
Intercept					<0.0001	3397.16	560.10
Slopes	Size*PA				<0.0001	41.46	4.37
	Size*PnoA				0.13	9.72	6.29
	Size*NoP				0.98	0.11	4.12

**Table 2:** Results of the multiple regression model testing for the effects of plant size and ant occupation rate on total leaf area.

Explanatory variables		*ndf*	*ddf*	*F*	*P*	Estimate	SE
Size		1	46	11.70	0.0013		
Occupation rate		1	46	20.46	<0.0001		
**Parameter estimates**							
Intercept					0.0147	2086.07	822.39
Slopes	Size				0.0013	16.04	4.69
	Occupation rate				<0.0001	7805.41	1725.56

**Table 3:** Results of the Poisson regression model testing for the effect of plant size, plant type (PA, PnoA and NoP) and their interaction on the number of functional leaves.

Explanatory variables	Plant type	*df*	*χ^2^*	*P*	Estimate	SE
Size		1	30.68	<0.0001		
Type		2	30.33	<0.0001		
**Parameter estimates**						
Intercepts	PA	1	865.28	<0.0001	2.5013^a^	0.0850
	PnoA	1	566.72	<0.0001	2.1251^b^	0.0893
	NoP	1	420.89	<0.0001	2.0064^b^	0.0978
Common slope	Size	1	33.01	<0.0001	0.0021	0.0004

The means that share the same letter were not statistically different according to the Wald's χ^2^ tests.

Foliar N concentration did not differ significantly between categories (Table 3, ANOVA). However, because mean foliar area differed between categories, and LMA (Leaf Mass Area) showed a similar, but non-significant tendency (*P* = 0.07, Table 4), total foliar N content will have differed between categories. For example, we estimated from the N concentration and LMA data (Table 4) and from the ANCOVA model on total leaf area (Fig. 2, Table 1), that a 175-cm plant occupied by *C. schmitzi* would show a 3.1- and a 3.4-fold increase in total foliar N content compared to equivalent-sized plants with unoccupied pitchers, and without pitchers, respectively (means: 1304, 424.2 and 382.2 mg, respectively).

**Table 4:** Effect of symbiont association and pitcher presence on leaf quality.

Leaf	PA	PnoA	NoP	*P*_1_	*P*_2_	*P*_3_
Nitrogen (%)	1.45±0.24^a^	1.40±0.17^a^	1.42±0.29^a^	0.85	0.23	0.47
Nutrient stress (SIPI)	0.995±0.011^a^	1.007±0.012^b^	0.999±0.010^ab^	0.02	0.82	0.88
Leaf area (cm^2^)	579.41±133.03^a^	381.24±138.62^b^	283.18±71.94^c^	<0.0001	0.10	0.34
LMA (g/m^2^)	83.59±11.27^a^	80.41±13.16^a^	73.55±13.28^a^	0.07	0.77	0.12

*P*_1_: Probabilities yielded by ANOVA.

*P*_2_: Probabilities yielded by Levene's test (homoscedasticity).

*P*_3_: Probabilities yielded by Shapiro's test (normality).

The values refer to means ±1 SE of each variable. Comparisons between plant categories were made by ANOVAs.

The means that share the same letters were not statistically different at *P*<0.05 according to the Ryan-Einot-Gabriel-Welsch multiple range tests.

Very few unoccupied plants had produced upper pitchers and entered into the climbing phase (Fig. 2). Indeed, of the 7 plants sampled that had begun to produce upper pitchers and thereby entered into this growth phase, only one was unoccupied by *C. schmitzi*. Other than this individual case, no similar plants were found at the study site.

#### Absence of measurable benefits provided by carnivory in plants not occupied by *C. schmitzi*

In the absence of *C. schmitzi*, pitcher-bearing *N. bicalcarata* plants did not show increased growth or production of photosynthetic surfaces, compared to conspecifics that lacked pitchers. There were no significant differences between PnoA plants and NoP plants in terms of foliar N concentration or LMA (Table 4). For leaf area, the PnoA plants showed significantly higher values than the NoP plants (Table 4). However, as there was no significant difference between the PnoA and NoP plants in the number of leaves (Table 3), this difference had no significant impact on the total leaf area at the plant scale, when the size effect was factored out (Fig. 2). Nutrient stress, as measured by the Structure Independent Pigment Index (SIPI), was significantly greater in PnoA plants than in PA plants, i.e., the absence of *C. schmitzi* induced nutrient stress in plants that produced pitchers. There was no significant difference in nutrient stress between PnoA and NoP plants, i.e., between plants with pitchers but not associated to *C. schmitzi* and those with no pitchers at all (Table 4). Taken together, these results demonstrate that, in the absence of its ant symbiont, *N. bicalcarata* appears to receive no measurable benefit from the production of pitchers.

### Possible mechanisms for the benefits conferred by *C. schmitzi*

**Figure 3:**
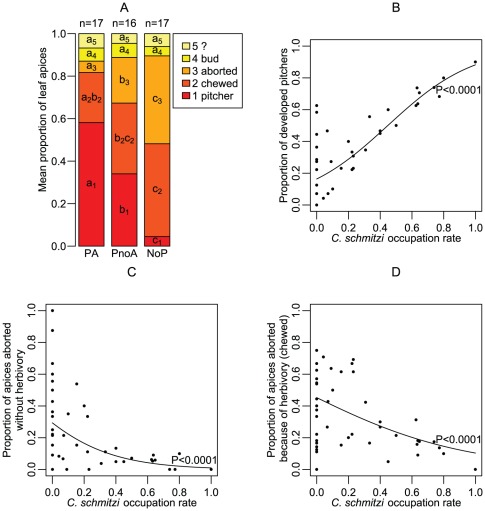
Positive effect *of Camponotus schmitzi* on pitcher production. (**a**) Mean proportions of apices that have remained buds, aborted, been chewed, developed into a pitcher or with unknown fate (?: tendril cut) compared for PA, PnoA and NoP plants. “Pitcher” refers to both living and dead pitchers (e.g., in NoP plants, the latter). (**b**) Proportion of functional pitchers as a function of *C. schmitzi* occupation rate. The line represents the estimated probability of an apex forming a pitcher, as predicted by logistic regression. (**c**) Proportion of apices that have aborted without herbivory as a function of *C. schmitzi* occupation rate. (**d**) Proportion of apices that have aborted because of herbivory as a function of *C. schmitzi* occupation rate.

The probability of a leaf apex producing a functional pitcher was significantly dependent on the status of the plant (being greater for PA plants; Fig. 3a, logistic regression, F_2,46_ = 49.92, *P*<0.0001). In addition, the probability of an apex producing a functional pitcher increased significantly with *C. schmitzi* occupation rate (logistic regression, F_1,47_ = 82.54, *P*<0.0001, Fig. 3b).

#### Prevention of pitcher abortion

This greater success in pitcher production shown by plants occupied by *C. schmitzi* is essentially linked to a lower rate of pitcher abortion (Fig. 3a). Indeed, whatever the cause of their abortion (herbivory damage referred to as “chewed”; other reasons, such as shortage of resources, merely referred to as “aborted”), the NoP plants showed higher rates of pitcher abortion than PnoA plants, which in turn showed higher rates of pitcher abortion than PA plants (Fig. 3a). In particular, the probability of a pitcher aborting for reasons other than herbivory decreased significantly with *C. schmitzi* occupation rate (logistic regression, F1,47 = 23.83 , P<0.0001, Fig. 3c).

#### Antiherbivore protection

The curculionid *Alcidodes* sp. was observed to chew the pitcher buds (Fig. 1). The mean proportion (± SE) of chewed buds for the PA plants (23.54±4.34%) was significantly lower than that of NoP plants (43.68±5.28%) but not that of PnoA plants (33.28±6.12%) (Fig. 3a). There was no statistical difference between PnoA and NoP plants, in the proportions of pitcher buds aborted due to herbivory, although the proportion of chewed buds decreased significantly with an increase in *C. schmitzi* occupation rate (logistic regression, significant effect of plant occupancy χ^2^ = 18.46, P<0.0001, Fig. 3d). Indeed, the logit model expressed a negative relationship between ant occupancy and the probability of a pitcher being damaged by chewing (estimate = −1.97, Waldχ^2^ = 16.36, P<0.0001).

There was no significant difference between the proportion of leaves damaged by herbivory for PA, PnoA, or NoP plants (21.22±3.92%, 24.40±5.91% and 25.81±4.88%, respectively; logistic regression, effect of plant category F_2,46_ = 0.31, *P* = 0.74). Herbivore pressure on the photosynthetic organs (i.e., the phyllodes) was weak (the leaves are tough and astringent, which denotes both mechanical and chemical protection). It was at least less important than that exerted on pitcher buds, as attested to by the mean proportion of herbivore-damaged leaves in NoP plants being less than half that of the mean proportion of damaged buds. The proportion of leaves damaged by herbivory did not show a significant decrease with *C. schmitzi* occupation rate (logistic regression, F_1,47_ = 1.65, *P* = 0.210). However, when only the plants that had been either previously, or were currently, occupied by *C. schmitzi* (identified by the presence of holes in domatia) were considered, the proportion of damaged leaves decreased significantly with plant occupancy (F_1,25_ = 29.90, *P*<0.0001). The logit model showed a negative relationship between *C. schmitzi* occupancy rate and the probability of a leaf being damaged by herbivores (estimate = −3.10, Waldχ^2^ = 25.23, *P*<0.0001).

#### Symbiont-augmented prey capture

**Figure 4:**
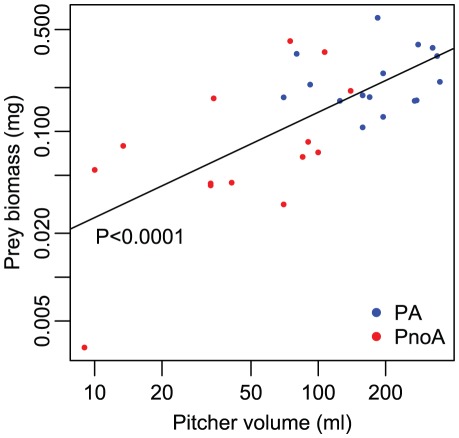
Positive effect of *Camponotus schmitzi* on pitcher volume and prey biomass. Prey biomass accumulated during the entire life of a lower pitcher as a function of pitcher volume (logarithmic scales). Blue and red points refer to PA and PnoA plants, respectively. A common regression line was estimated for these two categories, the means of which were significantly different.

Lower pitchers of plants occupied by *C. schmitzi* had a mean (± SE) prey biomass approximately twice that of pitchers of unoccupied plants (M_PA_ = 247.4±32.55 mg, n = 16; M_PnoA_ = 117.8±31.0 mg, n = 14). The ANOVA on the log-transformed data showed a significant effect of plant category (occupied *vs.* unoccupied) on prey biomass (R^2^ = 0.31, F_1,28_ = 12.79, *P* = 0.0013). However, this effect is essentially due to the positive effect of ant occupancy on pitcher volume. The logarithm of the prey biomass of lower pitchers increases linearly with the logarithm of pitcher volume (ANCOVA, R^2^ = 0.53, F_1,28_ = 26.89, *P<*0.0001, Fig. 4), regardless of whether or not the pitcher is occupied by *C. schmitzi* (no significant effects of the plant type*pitcher volume interaction: F_1,26_ = 1.89, *P* = 0.18 and of the plant type: F_1,27_ = 0.40, *P* = 0.53, i.e., the same slopes and intercepts). Pitcher volumes were significantly larger for plants occupied by *C. schmitzi* than for unoccupied plants (M_PA_ = 204.21±23.06 ml, M_PnoA_ = 59.99±10.93 ml; ANCOVA, R^2^ = 0.55, effect of plant type: F_1,28_ = 29.10, *P*<0.0001) and did not significantly increase with plant size (F_1,27_ = 0.31, *P* = 0.59), and not differently according to plant type (F_1,26_ = 1.99, *P* = 0.17).

The prey contents were dominated either by *Crematogaster* sp. (Formicidae, Myrmicinae, n = 15 pitchers), *Hospitalitermes* sp. (Termitidae, Nasutitermitinae, n = 10) or *Polyrhachis pruinosa* Mayr (Formicidae, Formicinae, n = 5). *P. pruinosa* was noticed to be more often dominant in upper pitchers which were, however, not taken into account in this study. There was no influence of *C. schmitzi* on the prevalence of *Crematogaster* sp. (present in large numbers in 14 out of the 16 lower pitchers inhabited by *C. schmitzi* and in 11 out of the 14 uninhabited ones, Fisher exact test, *P* = 0.64). There was also no influence of *C. schmitzi* on the prevalence of *Hospitalitermes* sp. (present in large numbers in 9 out of the 16 inhabited by *C. schmitzi* and in 7 out of the 14 uninhabited, Fisher exact test, *P* = 1). Neither was there an influence of *C. schmitzi* on the prevalence of *Polyrhachis* species other than *P. pruinosa* (present in 6 out of the 16 inhabited pitchers and in 6 out of the 14 uninhabited ones, Fisher exact test, *P* = 1). In contrast, there was a significant influence of *C. schmitzi* on the prevalence of *P. pruinosa*, the largest common prey item of *N. bicalcarata* (present with an average of 7 workers, min = 1, max = 41, in 13 of the 16 inhabited pitchers but in only 3 of the 14 uninhabited ones, mean = min = max = 2 workers, Fisher exact test, *P* = 0.0027). Moreover, *C. schmitzi* also had a significant influence on the prevalence of flying insects (present, albeit in small numbers (1–7 individuals) in all of the 16 inhabited pitchers but in only 5 of the 14 uninhabited ones, Fisher exact test, *P* = 0.0001), i.e., hymenopterans, dipterans and mainly (73%) coleopterans, which belonged to the phytophagous Families Chrysomelidae, Elateridae, Melolonthidae and Curculionidae, in decreasing order of numerical importance.

### The contribution of myrmecotrophy

#### *C. schmitzi* is ^15^N enriched compared to the main prey of *N. bicalcarata*

As expected for a species which feeds on the plant's prey, the mean δ^15^N of *C. schmitzi* was the highest (3.75±0.23‰), and differed significantly from those of *P. pruinosa* (1.52±0.17‰; difference in least square means: T = −7.80, *P*<0.0001) and *Hospitalitermes* sp. (−3.37±0.27‰, T = 20.17, *P*<0.0001), but not from that of *Crematogaster* sp. (3.35±0.62‰; T = −0.56, *P* = 0.58). The variances of the δ^15^N values of the various insect taxa being not homogenous (Levene's test F_3,40_ = 7.81, *P* = 0.0003), we used a linear model with unequal variances (SAS mixed procedure) to compare mean δ^15^N values.

#### Plants occupied by *C. schmitzi* are more ^15^N enriched than unoccupied plants

**Figure 5:**
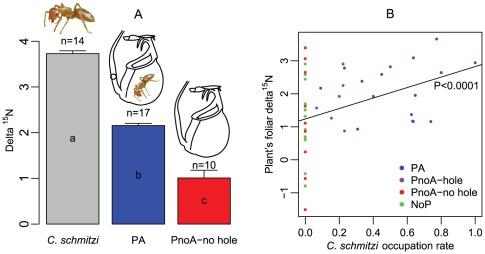
Isotopic signature (δ^15^N) of samples and assessment of myrmecotrophy. (**a**) Mean δ^15^N compared between *C. schmitzi*, plants occupied by *C. schmitzi* (referred as PA plants) and plants that have not been occupied by *C. schmitzi* (referred as PnoA_-no hole_). These three signatures were used to estimate the percentage of foliar nitrogen derived from the ants. The error bars denote 1 S.E. (**b**) Foliar δ^15^N of *N. bicalcarata* as an increasing function of *C. schmitzi* occupation rate. As a consequence the percentage of foliar N derived from the ants also increased with ant occupation rate.

δ^15^N differed significantly between plant categories (F_3,42_ = 3.10, P = 0.03). The mean δ^15^N of PA plants (2.16±0.19‰) was significantly higher than that of pitcher-producing plants that had never been occupied (PnoA-_no hole_, 1.01±0.53‰; difference of lsmeans: T = −2.02, P = 0.049, Fig. 5a). It was also significantly higher than that of plants without pitchers (NoP, 1.21±0.30‰, T = −2.62, P = 0.01), but did not differ significantly from plants that had been previously occupied, but were currently not (PnoA_-hole_, 1.79±0.30‰, T = −1.02, P = 0.31). As expected for plants capturing a variety of prey from different trophic levels, the unoccupied pitcher-bearing plants (PnoA_-no hole_) displayed the highest variance in δ^15^N. Because of N translocation from older to newer parts of the plant (see Materials and Methods), it was necessary to differentiate between PnoA plants that bore pitchers without any hole, and PnoA plants that bore some pitchers (max = 3) with a hole, denoting prior occupancy by *C. schmitzi*. The variances of foliar δ^15^N values of the different plant categories were not equal (Levene's test F_3,42_ = 5.09, P = 0.0043) and the means were thus compared taking into account the heterogeneity of variances.

#### The percentage of foliar N derived from *C. schmitzi* is a function of occupation rate

Foliar δ^15^N increased significantly with *C. schmitzi* occupation rate (F_1,44_ = 7.64, P = 0.0083, Fig. 5b). The amount of foliar N derived from *C. schmitzi*, estimated from the two-end member mixing model, was 42.14±14.28% (95% C.I. = 12.01–72.27%). When only PA plants with occupancy rates >75% were taken into account, the estimate increased to 76.09±12.57% (95% C.I. = 38.42–100%).

## Discussion

In this study, we provide evidence that *C. schmitzi* has a positive impact on the growth, N status and physiology of its carnivorous host-plant, *N. bicalcarata*. It is the first time that a carnivorous plant is thus shown to be engaged in a species-specific mutualism with an ant symbiont. *C. schmitzi* contributes substantially to the nutrition of *N. bicalcarata* via direct myrmecotrophy, as well as indirectly, by helping in capturing the prey and protecting the pitchers, which are the main N assimilatory organs. The relationship is thus a multifaceted nutritional mutualism. *N. bicalcarata* plants that deploy pitchers but lack the ant symbiont show reduced growth and N content, comparable to *N. bicalcarata* plants lacking pitchers entirely. The ant symbiont may therefore be a necessary factor in the plant's digestion process, and thus an integral component of the carnivorous syndrome in this plant.

### Evidence for a nutritional mutualism between the ant and its host plant

The benefits provided by the plants to the ants are three-fold. First, they provide a nest site, situated in the swollen and hollow pitcher tendrils [27]. Secondly, extrafloral nectar constitutes a carbohydrate resource, produced mainly by the two thorn-like glandular structures emanating from the apical teeth of the peristome (Fig. 1 [28]). Finally, the pitcher provides a reliable source of protein in the form of captured prey [26].

The benefits provided by the ants to the host have until now been either hypothetical or controversial. In the current study, we show that ant-occupied plants have a greater green leaf area, higher numbers of leaves, and higher foliar N content, than unoccupied plants of the same size. However, it could be argued that *C. schmitzi* simply choose the healthiest plants, and that the factors outlined above are a cause, rather than a consequence, of ant occupation. If this was the case, we would have expected a difference between *C. schmitzi*-occupied plants and unoccupied ones, but not a tight relationship between photosynthetic area and *C. schmitzi* occupation rate. As photosynthetic area increased gradually with the occupation rate, the size effect being taken into account, the correlation we observed is most likely the consequence of benefits conferred by the ant on its host plant.

A few studies of *Nepenthes* have shown that either prey addition (e.g. [43]) or prey deprivation [44] can have direct consequences on photosynthetic activity. The increase in total leaf area and total leaf N content displayed by ant-occupied plants therefore reflects a nutritional benefit provided by the ants. *Nepenthes* are more limited by N than by either P or K [45], which often co-limit photosynthetic activity in other carnivorous plants [46]. Many studies have shown that addition of prey enhances growth in several carnivorous plant taxa (see [46] for review). Hence the highest number of leaves produced by ant-occupied plants should partly be explained by enhanced growth. But the ant-occupied plants may also keep their leaves functional over a longer period. Earlier leaf abscissions, as observed in cases of nutrient stress [47], may merely explain the lowest number of functional leaves in uninhabited plants. *N. bicalcarata* displays by far the longest leaf lifespan of any *Nepenthes* species tested to date [48], [49]. This may be explained by the nutritional advantage conferred by the ant symbiont.

How do *C. schmitzi*-occupied plants obtain such a nutritional advantage? It was previously shown that the ants help the plant to retain prey once it has fallen into the pitcher [26]. Therefore the ant-occupied plants may gain a nutritional advantage merely because they capture more prey. However, as the ants also consume a proportion of the prey [26], [29], uncertainty remained regarding net nutritional benefit to the host plant. In the current study, we show at the pitcher level that consumption of prey by *C. schmitzi* is compensated for by the increase in prey amount resulting from its ambush behaviour: there was no difference in prey biomass accumulated by pitchers of occupied and unoccupied plants, when pitcher size was taken into account. This result demonstrates that *C. schmitzi* is not a kleptoparasite (i.e., it does not steal prey from the plant). It also indicates that the nutritional benefit conferred by the ants is due to two factors: (1) a greater total volume of traps, i.e., more pitchers (2.8-fold), of larger size, with consequently greater prey biomass (approximately double); and (2) myrmecotrophy, i.e., *C. schmitzi* consumes and digests prey, and the nutrients are transferred to the host plant via *C. schmitzi* feces and carcases. The recent finding by Thornham *et al.* [35] that *C. schmitzi* increases the trapping efficiency of *N. bicalcarata* by maintaining the slipperiness of the peristome, adds another facet to the story. However if the cleaning behaviour of *C. schmitzi* was the main mechanism explaining enhancement of prey capture, one would expect a similar increase of prey capture on all categories of prey. Here we observed that the increase in prey capture concerns only large prey items such as *P. pruinosa* or flying insects, i.e., insects that can use their wings to cope with the trap. It did not concern more easily-caught prey such as small *Crematogaster* sp. or termites, whose initial capture should have been affected by the supposedly greater slipperiness of the trap. The retention of these small prey items is due to an inability to extricate themselves from the fluid once trapped [26]. Thus, the ambush hunting behaviour of *C. schmitzi* ([26] on-line video clip, http://umramap.cirad.fr/amap3/cm/index.php?page=films-2) is the more likely mechanism explaining the retention and prevalence of large or flying prey items in the inhabited pitchers.

Why do *C. schmitzi*-occupied plants have more functional pitchers? *C. schmitzi* may reduce the rate of pitcher abortion by protecting the pitcher buds against herbivores, as suggested by the decreased incidence of chewed apices with increased *C. schmitzi* occupancy. This confirms previous findings with regards to protection against bud-chewing *Alcidodes* sp. weevils [30]. Similar findings have been reported in the American pitcher plant *Sarracenia minor* Walter where the nectar-visiting ants, which also constitute prey of the plant, were shown to protect pitchers against herbivory [50]. However, in *N. bicalcarata* almost half of the aborted pitchers do so for reasons other than herbivory, especially in plants not occupied by *C. schmitzi.* Hence, other factors may contribute to the lower abortion rate. For NoP plants, it could be argued that the soil in the immediate vicinity of the roots might contain sufficient available N, and these plants do not need to produce expensive pitchers to augment N uptake, as demonstrated by the results of fertilization experiments carried out in other pitcher plants [51]. However, given their weak leaf surface and growth performance, these plants were most probably N-limited and their photosynthate production was consequently not sufficient to allow for construction of resource-demanding traps.

### The benefits of myrmecotrophy and the importance of the digestion service provided by the ants

As confirmed by the elevated isotopic signature of its tissues (δ^15^N values in animal tissue increase at each trophic level [39], [40]), *C. schmitzi* is an insect predator, probably the top predator of the food web found within *N. bicalcarata* pitchers [22]. Its mean δ^15^N value was higher than those reported for congeners in Brunei [4], reflecting a more carnivorous diet which may be a result of coevolution with a carnivorous plant species. As *C. schmitzi*-occupied plants had higher foliar δ^15^N values than unoccupied ones, and as δ^15^N reflects diet, we can conclude, without much doubt, that *C. schmitzi* directly feed the host plant with their δ^15^N-elevated remains and feces, which were observed to fall into the pitchers. It could be argued that the more elevated δ^15^N values in plants occupied by the symbiont have nothing to do with myrmecotrophy but instead reflect a *C. schmitzi*-induced change in prey composition in favour of prey with higher tissue δ^15^N values. However, this was not the case. Indeed, as indicated by our analysis of pitcher contents, *C. schmitzi* occupation results in higher occurrence of prey with relatively low δ^15^N values, such as the nectar-feeding *P. pruinosa* and phytophagous coleopterans. Therefore, the myrmecotrophy hypothesis is the most likely. Furthermore, our estimate is conservative since, because of this possible bias, it likely underestimates the percentage of foliar N derived from the ants. Two main results further attest to a flux of N from *C. schmitzi* to *N. bicalcarata*. The plants capture large numbers of termites, the tissues of which show negative δ^15^N values (−3.37±0.27‰) which may explain occasional negative foliar δ^15^N values observed only in uninhabited plants. Despite this, foliar δ^15^N values of inhabited *N. bicalcarata* (2.15±0.20‰) are the highest recorded from any *Nepenthes* species analyzed to date from Brunei (*N. rafflesiana* var. *typica* Jack, primarily ant-fed = 1.9±0.14‰, [52]; *N. rafflesiana* var. *elongata* Hort, a putative bat feces specialist = 1.30±1.53‰, [53]; *N. albomarginata* T. Lobb *ex* Lindl, a termite trapper = −2.1±0.31‰, [52]; *N. ampullaria* Jack, a species that derives >30% of its N from leaf litter = −2.22±0.19‰; [54]). The second finding that strongly supports the hypothesis of an N flux from *C. schmitzi* to *N. bicalcarata* is that foliar δ^15^N values increase significantly with *C. schmitzi* occupation rate as would be expected if the plant derived a significant part of its N directly from the ants.

In typical myrmecotrophic ant-plant systems, the plants receive N from ant waste accumulated within the domatia (e.g. [12], [13]). During our dissection of more than 30 *N. bicalcarata* domatia, we observed no ant wastes, nor any chambers specifically devoted to such material. Nor did we observe the presence of any fungi, which have been shown to facilitate assimilation of ant remains in other myrmecotrophic systems [14], [15]. This implies that the feces fall directly into the pitchers, which are already adapted for nutrient uptake. Putative fecal particles were observed in the pitcher fluid; in addition, 100% of *N. bicalcarata* pitchers were observed to contain *C. schmitzi* carcases. These carcases included queens killed in intra-specific fights for domatia colonisation, as well as workers that died naturally or in the course of attacking prey. But given the relatively small number of *C. schmitzi* carcases compared to the prey items of other species, the fecal contribution of *C. schmitzi* to the plant's N input is likely to be more important than its direct contribution as prey. We estimated 42.14±14.28% of *N. bicalcarata*'s foliar N to be derived from *C. schmitzi* waste material, a figure that rises to 76.09±12.57% for the most heavily-colonized plants.

Myrmecotrophy is therefore important in this ant-plant system, compared to other, sympatric non-carnivorous ant-plants systems such as the ant-epiphyte *Dischidia major* (Vahl) Merr. (Asclepiadaceae), which derives only 29% of its N from ants [12]. This higher myrmecotrophic output might be attributed to the carnivorous habit of the plant, which implies a whole body of adaptations that should favour insect nutrient-derived uptake.

But is *N. bicalcarata* a “true” carnivorous plant? What is its carnivorous efficiency in the absence of *C. schmitzi*? This question appears legitimate since *N. bicalcarata* differs from more “typical” congeners in several carnivorous traits, including the absence of a waxy layer in the pitcher, the presence of a non-viscoelastic fluid [31], and a weakly acidic fluid [26], [55]. The fact that pitcher-producing plants that were not occupied by *C. schmitzi* did not produce a greater total leaf area than plants without any pitchers suggests that assimilation of insect-derived nutrients in these plants is insufficient to offset the costs of pitcher production. Adult *Nepenthes* plants depend chiefly on the nutrients derived from the prey [44], [56] which may explain why we were unable to find unoccupied plants that had produced upper (aerial) pitchers and thus entered the adult, climbing phase.

Does *N. bicalcarata* depend entirely on its ant symbiont to digest and assimilate prey? It has been proposed that the exceptionally rich infauna associated with the digestive fluid of *N. bicalcarata* is responsible for prey breakdown [22], an idea also suggested for phytotelmata-associated plants in general [21]. But to date only *C. schmitzi* has been shown experimentally to be implicated in prey breakdown [26]. In the absence of the symbiont, prey items remained intact 15 days after addition to *N. bicalcarata* pitchers; in contrast, prey breakdown was rapid in the presence of *C. schmitzi* [26]. The relatively high pH (i.e. low acidity) of the digestive fluid in this species (*ca.* 5; [26], [48]) might reduce the rate of prey breakdown. Indeed, the digestive enzymes Nepenthesin I and II, which are usually active at pH 2 to 3 [57], might be either absent or inactive in *N. bicalcarata*. Perhaps digestion is partly undertaken by bacteria, as in other carnivorous plants (e.g. the Sarraceniaceae and some protocarnivorous plants [18]), but enzymatic activity within the digestive tract of *C. schmitzi* itself, which transforms the prey into easily-assimilated feces, is undoubtedly beneficial to the plant. Hence the ant symbiont might be thought of as the “gizzard” of its carnivorous host. Pseudo-carnivorous plants that need a third partner for prey digestion have been identified previously [58]. For instance, it has been demonstrated in the South African *Roridula gorgonias* (Roridulaceae), the prey of which is digested by the mutualistic bug *Pameride roridulae* (Miridae), which then defecates onto the foliar surface [59].

Finally, in addition to enhancing nutrient assimilation by the pitcher, *C. schmitzi* may also limit nutrient loss from the pitcher. It can indeed be argued that this species, which is the top predator of the fluid-associated microecosystem, could contribute indirectly to the N flux of occupied plants by reducing adult emergence and escape of detritivores or intermediate predators. This has been elegantly demonstrated in experiments on the top predators of a bromeliad's phytotelm [60]. By preying upon mosquito larvae, for instance, as casually observed, the ants would thereby limit nutrient export from the system.

In conclusion, the results of the morphological, chemical, isotopic and reflectance analyses all point to *N. bicalcarata* being involved in a multifaceted nutritional mutualism with *C. schmitzi*. Because it seems to have lost some of the classical features involved in the carnivorous syndrome (absence of slippery wax and viscoelastic fluid, reduced acidity, putative absence of functional enzymes), *Nepenthes bicalcarata* may be viewed as a plant with an intermediate strategy between mutualism and antagonism. The ant symbiotic association is likely to have promoted a reduced harmful capacity in the antagonistic partner, i.e. the carnivorous plant, as expected by the theory of coevolution in some parasitic systems [61]. Nevertheless, if the plant's capacity to harm its specific ant symbiont has indeed been reduced by selection, we have also demonstrated that the association with the symbiotic ants represents an advantageous substitute for the carnivorous plant because it increases its efficiency as a predator of other insects (resulting in plants with more traps and more efficient trapping strategy). Therefore, this paradoxical but synergistic combination of carnivory and ant-plant mutualism results in a highly efficient nutrient sequestration strategy. This may explain why *Nepenthes bicalcarata* displays exceptional leaf life span and vegetative growth, reaching heights up to 20 m into the forest canopy [20], a record for the genus. Finally, this mixed strategy represents an outstanding adaptation for the exploitation of nutrient-poor soils and is, to our knowledge, unique in the plant kingdom.

## Materials and Methods

### Study site

The study was carried out in peat swamp forest in Brunei, NW Borneo (04°33′N. 114°29′E, 35–45 m asl), in February 2011. The habitat was dominated by *Shorea albida* Symington (Dipterocarpaceae). The most common *Nepenthes* were *N. bicalcarata* and *N. ampullaria.* Light-demanding congeners such as *N. gracilis* Korth and *N. rafflesiana* var. *elongata* were present in areas in which the canopy had been opened due to timber extraction.

At this site, approximately 70% of *N. bicalcarata* were pitcher-bearing ant-inhabited plants (PA), 25% were pitcher-bearing uninhabited plants (PnoA) and 5% were pitcher-lacking plants (NoP). When considering only adult plants, the proportion of PA plants was even higher.

### Sampling procedures

We sampled 50 *N. bicalcarata* plants (with a total of 617 fully-developed leaves) in order to obtain 17 plants bearing pitchers inhabited by *C. schmitzi* (PA), 16 plants with uninhabited pitchers (PnoA), and 17 plants with no living pitchers (NoP), ranging in height from 20 to 450 cm. Hence for comparative purposes and statistical reliability, our sample was intentionally biased (compared to the relative frequencies occurring naturally in the population) to obtain three subsamples of approximately equal numbers of plants. Among plants that bore pitchers, seven had upper pitchers (6 PA and 1 PnoA) and had entered into their mature, climbing phase. PnoA plants observed to be unoccupied at the time of sampling may have been formerly occupied by *C. schmitzi*; the presence of a hole within the swollen but empty pitcher tendril provided evidence of this in six plants. Not infrequently, inundation events remove *C. schmitzi* colonies from lower pitchers. In the same manner, NoP plants observed without any pitchers may have borne pitchers in the past, some of which may have housed *C. schmitzi* colonies. In four plants, the most basal leaf bore the remains of dried, swollen tendrils that had been hollowed out.

For each plant, every leaf was numbered, measured (length and width) and assessed for herbivory. The apex of each tendril was scored under one of five categories: “pitcher”, “bud”, “chewed”, “aborted” and “cut”, which refer respectively to opened functional pitcher, growing pitcher bud, apex aborted due to having been chewed, apex aborted for any other reason, and unknown apex state (cut tendril). For each plant, we defined *C. schmitzi* occupation rate as the total number of tendrils with holes, divided by the total number of green leaves. The most recent fully-developed leaf of each plant (n = 50) was removed, photographed and used for isotopic and nutrient stress analyses. The most basal and still- functioning lower pitcher of each plant (n = 30) was selected for a prey biomass comparison Data were analysed using R v. 2.10.1 (www.r-project.org) and SAS v. 9.2 (SAS Institute Inc., Cary, NC).

### Estimation of leaf area, LMA, pitcher volume and prey biomass

Each leaf was measured for width and length (n = 617). The area of each removed leaf (n = 50) was assessed from digital photographs using the Toaster plug-in for ImageJ v. 1.44p (http://rsbweb.nih.gov/ij/index.html). The logarithm of leaf area was found to be a linear function of the logarithms of its length and width: (log(Leaf area) = −0.78+1.13* log(Length)+0.96*log(Width), F_2,46_ = 795.5, R^2^ = 0.97, *P*<0.0001). We were thus able to estimate accurately the leaf area for the 567 remaining leaves. The total leaf area of each plant was then estimated. We also measured the leaf mass per unit area (LMA) of the 50 removed leaves by weighing an 18 mm-diameter disk punched out from the dry surface of each.

For each open pitcher (n = 174), we measured the maximum height (dorsal face; measurement L1), the minimum height (ventral face; measurement L2) and the maximum diameter of the peristome (measurement L3), respectively. Prey contents of the selected lower pitchers (n = 30) were collected using a filter, dried at 60°C for 72 h, then weighed on an analytical balance (precision ±0.1 mg). We measured the volumes of the emptied lower pitchers (n = 30) by completely filling them with water and gauging the water volume using a burette. The logarithm of pitcher volume was found to be a linear function of the logarithms of measurements L1, L2 and L3: (log(pitcher volume) = −0.17−0.13*log(L1)+0.94*log(L2)+2.42*log(L3), F_3,29_ = 232.3, R^2^ = 0.96, *P*<0.0001). We were therefore able to estimate accurately the volume of each of the remaining 141 pitchers.

### Analysis of leaf herbivory and pitcher abortion

We analysed, using logistic regressions, the proportions of leaves damaged by herbivory, pitcher buds aborted due to herbivory, i.e. with damages by chewing insects, and pitcher buds aborted for unknown reasons, i.e. observed to be dessicated and no longer functional. For this, we used the GENMOD procedure in SAS, correcting when necessary for data overdispersion using the ratio of Pearson's χ^2^ to the associated number of degrees of freedom. We first tested for the effect of plant category (NoP, PnoA, PA) on the dependent variable, and then tested for the effect of the quantitative variable (*C. schmitzi* occupancy rate).

### Nutrient stress analysis via foliar reflectance

We undertook normal reflectance scans (*ca.* 0.5 cm^2^ in area) of the adaxial surface of the phyllode of each freshly-removed leaf. The scans were conducted in natural, indirect light at 1-nm intervals from 400 nm (blue) to 800 nm (near infra red) using a model USB4000 spectroradiometer (Ocean Optics Inc., Dunedin, FL) and fibre optic probe (BIF200-UV/VIS, Ocean Optics). Two scans were taken per leaf, at the mid-point on either side of the central rib, and averaged. After each leaf reflectance measurement, a scan was taken of a Spectralon® white standard (WS-1, Ocean Optics). Leaf reflectance values were divided by the white standard values to provide a normalized reflectance index. Prior to normalization, the dark signal was subtracted from each reflectance measurement. For each sample, we derived the Structure Independent Pigment Index (SIPI) as follows:
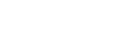
where *R* is the reflectance value at the wavelength in subscript [37]. SIPI increases with nutrient stress [37], [38]. Reflectance indices such as the Photochemical reflectance Index (PRI), which relies on xanthophyll cycle pigments [62], and reflectance in the green waveband (which relies on foliar chlorophyll concentration alone; [63]) are sensitive both to nutrient status and ambient light intensity. In contrast, the SIPI, which provides a proxy estimate of the ratio of total carotenoids to chlorophyll *a* [37], is not influenced by changes in light intensity [38]. Consequently, it can be used to compare relative nutrient stress of plants in the heterogeneous light environment of the forest understory.

### Analyses of pitcher contents, N concentration and isotopic composition

Discrimination against heavy stable isotopes results in predictable patterns of isotopic distribution within ecosystems; in the case of δ^15^N, enrichment in animal tissues is between 3 and 5‰ per trophic level [39], [40]. *C. schmitzi* was shown to be a predator of insects that had fallen into the pitchers [22], [26]. We analysed the prey contents of 30 pitchers using a binocular microscope. We first noticed that *C. schmitzi* carcases were present in each inhabited pitcher. We also noticed that *Crematogaster* sp. (a nectar-feeding but also carnivorous ant [4]), *P. pruinosa* (a mainly nectar and epiphyll-eating ant [4]), and *Hospitalitermes* sp. (lichen-eating termites [4]) were the main prey of *N. bicalcarata* at this location. To verify the predatory status (via trophic position) of *C. schmitzi*, we then determined δ^15^N values for *C. schmitzi* and its most probable prey items, i.e. the three species above. If δ^15^N values for *C. schmitzi* were significantly elevated relative to the prey species, and if *C. schmitzi*-inhabited *N. bicalcarata* plants were found to have higher tissue δ^15^N values than uninhabited plants, such findings would support the myrmecotrophy hypothesis (i.e., that *C. schmitzi* material (carcases, feces) contributes to the N budget of *N. bicalcarata*). Ant and termite workers from discrete colonies (*C. schmitzi*, n = 14; *Crematogaster* sp., n = 10; *P. pruinosa*, n = 10 and *Hospitalitermes* sp., n = 10) were collected from separate *N. bicalcarata* plants using an aspirator, then killed by freezing. Approximately 5 mg of each sample was oven-dried at 60°C for 8 h, then pulverised. As young *Nepenthes* leaves are major sinks for prey-derived N acquired by older pitchers [56], and because isotopic signature may vary with leaf age, we selected the most recent fully-developed leaf from each plant (n = 50). The assumption of translocation of prey-derived N to young leaves was supported by the higher correlation between insect biomass captured by a pitcher and the area of the leaf produced at the next node (r_Pearson_ = 0.55, *P* = 0.002, n = 30) than that between insect biomass and leaf area of the same node (r_Pearson_ = 0.49, *P* = 0.006, n = 30). Four samples belonging to NoP plants bore traces of moisture despite relatively quick drying and were thus not used for isotopic analyses. Isotopic abundances were measured using an isotope ratio mass spectrometer (Delta V Plus thermo-coupling microanalyzer (CN) FLASH EA 1112 and Delta S coupling microanalyzer (NC) SCA model) at the Service Central d'Analyse of the CNRS (SCA, Solaize, France).

The contribution of *C. schmitzi* material to foliar N in *N. bicalcarata* was estimated using the classical two-end member mixing model [42]:
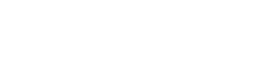
where δ^15^N_PA_, δ^15^N_PnoA-no hole_ and δ^15^N*_C. schmitzi_* refer to the mean δ^15^N of the samples from *C. schmitzi*-inhabited plants, uninhabited plants and *C. schmitzi*, respectively. For this analysis, we ensured that the PnoA plants showed no trace (hole in domatia) of prior occupation by *C. schmitzi*. The N analysis also yielded N concentration values, which in combination with the morphometric data, allowed estimation of total N content of the leaves of individual plants.

## References

[1] McKey D, Gaume L, Brouat C, Di Giusto B, Pascal L, Burslem D, Pinard M, Hartley S (2005). The trophic structure of tropical ant-plant-herbivore interactions: community consequences and coevolutionary dynamics.. Biotic Interactions in the Tropics: Their Role in the Maintenance of Species Diversity.

[2] Bronstein JL, Alarcón R, Geber M (2006). The evolution of plant-insect mutualisms.. New Phytologist.

[3] Gaume L, McKey D, Terrin S (1998). Ant-plant-homopteran mutualism: how the third partner affects the interaction between a plant-specialist ant and its myrmecophyte host.. Proceedings of the Royal Society B: Biological Sciences.

[4] Davidson D, Cook SC, Snelling RR, Chua TH (2003). Explaining the abundance of ants in lowland tropical rainforest canopies.. Science (New York, NY).

[5] Heil M, McKey D (2003). Protective Ant-Plant Interactions As Model Systems in Ecological and Evolutionary Research.. Annual Review of Ecology, Evolution, and Systematics.

[6] Rico-Gray V, Oliveira PS (2007). The Ecology and Evolution of Ant-Plant Interactions.

[7] Beattie A (1989). Myrmecotrophy: plants fed by ants.. Trends in Ecology & Evolution.

[8] Benzing DH, Huxley CR, Cutler DF (1991). Myrmecotrophy: origins, operation, and importance.. Ant-plant Interactions.

[9] Rickson FR (1979). Absorption of animal tissue breakdown products into a plant stem-the feeding of a plant by ants.. American Journal of Botany.

[10] Rico-Gray V, Barber JT, Thien LB, Ellgaard EG, Toney JJ (1989). An unusual animal-plant interaction: feeding of *Schomburgkia tibicinis* (Orchidaceae) by ants.. American Journal of Botany.

[11] Gay H (1993). Animal-fed plants: an investigation into the uptake of ant-derived nutrients by the far-eastern epiphytic fern *Lecanopteris* Reinw.(Polypodiaceae).. Biological Journal of the Linnean Society.

[12] Treseder K, Davidson D, Ehleringer JR (1995). Absorption of ant-provided carbon dioxide and nitrogen by a tropical epiphyte.. Nature.

[13] Solano PJ, Dejean A (2004). Ant-fed plants: comparison between three geophytic myrmecophytes.. Biological Journal of the Linnean Society.

[14] Defossez E, Djiéto-Lordon C, McKey D, Selosse M-A, Blatrix R (2011). Plant-ants feed their host plant, but above all a fungal symbiont to recycle nitrogen.. Proceedings Biological sciences/The Royal Society.

[15] Leroy C, Séjalon-Delmas N, Jauneau A, Ruiz-González M-X, Gryta H (2011). Trophic mediation by a fungus in an ant-plant mutualism.. Journal of Ecology.

[16] Thompson JN (1981). Reversed animal-plant interactions: the evolution of insectivorous and ant-fed plants.. Biological Journal of the Linnean Society.

[17] Benzing DH (1987). The origin and rarity of botanical carnivory.. Trends in Ecology & Evolution.

[18] Juniper B, Robins R, Joel DM (1989). The carnivorous plants.

[19] Ellison AM, Gotelli NJ, Brewer JS, Cochran-Stafira DL, Kneitel JM (2003). The evolutionary ecology of carnivorous plants.. Advances in Ecological Research.

[20] Clarke C (1997). *Nepenthes* of Borneo.

[21] Beaver R, Frank JH, Lounibos LP (1983). The communities living in *Nepenthes* pitcher plants: fauna and food webs.. Phytotelmata: Terrestrial Plants as Hosts of Aquatic Insect Communities.

[22] Clarke C, Kitching R (1993). The metazoan food webs from six Bornean *Nepenthes* species.. Ecological Entomology.

[23] Cheek M (2001). Nepenthaceae.. Flora Malesiana.

[24] Gaume L, Di Giusto B (2009). Adaptive significance and ontogenetic variability of the waxy zone in *Nepenthes rafflesiana*.. Annals of Botany.

[25] Mcpherson S (2009). Pitcher Plants of the Old World Vol. One. Red Fern Natural History Production.

[26] Bonhomme V, Gounand I, Alaux C, Jousselin E, Barthélémy D (2011). The plant-ant *Camponotus schmitzi* helps its carnivorous host-plant *Nepenthes bicalcarata* to catch its prey.. Journal of Tropical Ecology.

[27] Beccari O (1885). Plantes à fourmis de l'Archipel Indo-Malais et de la Nouvelle Guinée.. Arch Ita Biol.

[28] Merbach MA, Zizka G, Fiala B, Merbach D, Maschwitz U (1999). Giant nectaries in the peristome thorns of the pitcher plant *Nepenthes bicalcarata* Hooker f.. Ecotropica.

[29] Clarke C, Kitching R (1995). Swimming ants and pitcher plants: a unique ant-plant interaction from Borneo.. Journal of Tropical Ecology.

[30] Merbach MA, Zizka G, Fiala B, Merbach D, Booth WE (2007). Why a carnivorous plant cooperates with an ant–selective defense against pitcher-destroying weevils in the myrmecophytic pitcher plant *Nepenthes bicalcarata* Hook. f.. Ecotropica.

[31] Bonhomme V, Pelloux-Prayer H, Jousselin E, Forterre Y, Labat J-J (2011). Slippery or sticky? Functional diversity in the trapping strategy of *Nepenthes* carnivorous plants.. New Phytologist.

[32] Gaume L, Perret P, Gorb EV, Gorb SN, Labat J-J (2004). How do plant waxes cause flies to slide? Experimental tests of wax-based trapping mechanisms in three pitfall carnivorous plants.. Arthropod Structure & Development.

[33] Gaume L, Forterre Y (2007). A viscoelastic deadly fluid in carnivorous pitcher plants.. PloS one.

[34] Bohn HF, Federle W (2004). Insect aquaplaning: *Nepenthes* pitcher plants capture prey with the peristome, a fully wettable water-lubricated anisotropic surface.. Proceedings of the National Academy of Sciences, USA.

[35] Thornham DG, Smith JM, Ulmar Grafe T, Federle W (2011). Setting the trap: cleaning behaviour of *Camponotus schmitzi* ants increases long-term capture efficiency of their pitcher plant host, *Nepenthes bicalcarata*.. Functional Ecology.

[36] Carter GA (1994). Ratios of leaf reflectances in narrow wavebands as indicators of plant stress.. International Journal of Remote Sensing.

[37] Peñuelas J, Baret F, Filella I (1995). Semi-empirical indices to assess carotenoids/chlorophyll a ratio from leaf spectral reflectance.. Photosynthetica.

[38] Moran JA, Mitchell AK, Goodmanson G, Stockburger KA (2000). Differentiation among effects of nitrogen fertilization treatments on conifer seedlings by foliar reflectance: a comparison of methods.. Tree Physiology.

[39] Deniro M, Epstein S (1981). Influence of diet on the distribution of nitrogen isotopes in animals.. Geochimica et Cosmochimica Acta.

[40] Minagawa M, Wada E (1984). Stepwise enrichment of 15N along food chains: Further evidence and the relation between δ ^15^N and animal age.. Geochimica et Cosmochimica Acta.

[41] Shearer G, Kohl DH, Rundel PW, Ehleringe JR, Nagy KA (1988). δ ^15^N method of estimating N_2_ fixation.. Stables Isotopes in Ecological Research.

[42] Phillips DL, Gregg JW (2001). Uncertainty in source partitioning using stable isotopes.. Oecologia.

[43] Pavlovic A, Singerová L, Demko V, Hudák J (2009). Feeding enhances photosynthetic efficiency in the carnivorous pitcher plant *Nepenthes talangensis*.. Annals of Botany.

[44] Moran JA, Moran AJ (1998). Foliar reflectance and vector analysis reveal nutrient stress in prey-deprived pitcher plants (*Nepenthes rafflesiana*).. International Journal of Plant Sciences.

[45] Osunkoya OO, Daud SD, Di Giusto B, Wimmer FL, Holige TM (2007). Construction costs and physico-chemical properties of the assimilatory organs of *Nepenthes* species in Northern Borneo.. Annals of Botany.

[46] Ellison AM (2006). Nutrient limitation and stoichiometry of carnivorous plants.. Plant Biology (Stuttg).

[47] Feller U, Anders I, Mae T (2008). Rubiscolytics: fate of Rubisco after its enzymatic function in a cell is terminated.. Journal of Experimental Botany.

[48] Clarke C (1998). Initial colonisation and prey capture in *Nepenthes bicalcarata* (Nepenthaceae) pitchers in Brunei.. Sandakania.

[49] Osunkoya OO, Daud SD, Wimmer FL (2008). Longevity, lignin content and construction cost of the assimilatory organs of *Nepenthes* species.. Annals of Botany.

[50] Moon DC, Rossi AM, Depaz J, McKelvey L, Elias S (2010). Ants provide nutritional and defensive benefits to the carnivorous plant *Sarracenia minor*.. Oecologia.

[51] Ellison AM, Gotelli NJ (2002). Nitrogen availability alters the expression of carnivory in the northern pitcher plant, *Sarracenia purpurea*.. Proceedings of the National Academy of Sciences USA.

[52] Moran JA, Merbach MA, Livingsont NJ, Clarke C, Booth WE (2001). Termite prey specialization in the pitcher plant *Nepenthes albomarginata*—evidence from stable isotope analysis.. Annals of Botany.

[53] Grafe TU, Schöner CR, Kerth G, Junaidi A, Schöner MG (2011). A novel resource-service mutualism between bats and pitcher plants.. Biology Letters.

[54] Moran JA, Clarke C, Hawkins BJ (2003). From Carnivore to Detritivore? Isotopic Evidence for Leaf Litter Utilization by the Tropical Pitcher Plant *Nepenthes ampullaria*.. International Journal of Plant Sciences.

[55] Moran JA, Hawkins BJ, Gowen BE, Robbins SL (2010). Ion fluxes across the pitcher walls of three Bornean *Nepenthes* pitcher plant species: flux rates and gland distribution patterns reflect nitrogen sequestration strategies.. Journal of Experimental Botany.

[56] Schulze W, Schulze ED, Pate JS, Gillison AN (1997). The nitrogen supply from soils and insects during growth of the pitcher plants *Nepenthes mirabilis*, *Cephalotus follicularis* and *Darlingtonia californica*.. Oecologia.

[57] Athauda SBP, Matsumoto K, Rajapakshe S, Kuribayashi M, Kojima M (2004). Enzymic and structural characterization of nepenthesin, a unique member of a novel subfamily of aspartic proteinases.. Biochemical Journal.

[58] Anderson B (2003). Digestive mutualism, an alternate pathway in plant carnivory.. Oikos.

[59] Ellis A, Midgley J (1996). A new plant-animal mutualism involving a plant with sticky leaves and a resident hemipteran insect.. Oecologia.

[60] Ngai JT, Srivastava DS (2006). Predators accelerate nutrient cycling in a bromeliad ecosystem.. Science.

[61] Combes C (1995). Parasitism: the Ecology and Evolution of Intimate Interactions..

[62] Gamon J, Serrano L (1997). The photochemical reflectance index: an optical indicator of photosynthetic radiation use efficiency across species, functional types, and nutrient levels.. Oecologia.

[63] Jacquemoud S, Baret F (1990). PROSPECT: A model of leaf optical properties spectra.. Remote Sensing of Environment.

